# A Data Mining Metabolomics Exploration of Glaucoma

**DOI:** 10.3390/metabo10020049

**Published:** 2020-01-28

**Authors:** Judith Kouassi Nzoughet, Khadidja Guehlouz, Stéphanie Leruez, Philippe Gohier, Cinzia Bocca, Jeanne Muller, Odile Blanchet, Dominique Bonneau, Gilles Simard, Dan Milea, Vincent Procaccio, Guy Lenaers, Juan M. Chao de la Barca, Pascal Reynier

**Affiliations:** 1 Faculté de Santé, Institut MITOVASC, UMR CNRS 6015, INSERM U1083, Université d’Angers, 49933 Angers, France; cinzia.bocca@univ-angers.fr (C.B.); DoBonneau@chu-angers.fr (D.B.); viprocaccio@chu-angers.fr (V.P.); guy.lenaers@inserm.fr (G.L.); JMChaoDeLaBarca@chu-angers.fr (J.M.C.d.l.B.); 2 Faculté de Pharmacie de Paris, CiTCoM UMR 8038 CNRS, Université Paris Descartes, 75270 Paris, France; 3 Département d’Ophtalmologie, Centre Hospitalier Universitaire, 49933 Angers, France; Khadidja.Boutellis@chu-angers.fr (K.G.); stephanieleruez@hotmail.fr (S.L.); PhGohier@chu-angers.fr (P.G.); Jeanne.Muller@chu-angers.fr (J.M.); 4 Centre de Ressources Biologiques, BB-0033-00038, Centre Hospitalier Universitaire, 49933 Angers, France; OdBlanchet@chu-angers.fr; 5 Département de Biochimie et Génétique, Centre Hospitalier Universitaire, 49933 Angers, France; gisimard@chu-angers.fr; 6 Singapore Eye Research Institute, Singapore National Eye Centre, Duke-NUS, Singapore 168751, Singapore; dan.milea@snec.com.sg

**Keywords:** data mining, metabolomics, mitochondrial dysfunction, optic neuropathy, primary open-angle glaucoma

## Abstract

Glaucoma is an age related disease characterized by the progressive loss of retinal ganglion cells, which are the neurons that transduce the visual information from the retina to the brain. It is the leading cause of irreversible blindness worldwide. To gain further insights into primary open-angle glaucoma (POAG) pathophysiology, we performed a non-targeted metabolomics analysis on the plasma from POAG patients (*n* = 34) and age- and sex-matched controls (*n* = 30). We investigated the differential signature of POAG plasma compared to controls, using liquid chromatography coupled to high resolution mass spectrometry (LC-HRMS). A data mining strategy, combining a filtering method with threshold criterion, a wrapper method with iterative selection, and an embedded method with penalization constraint, was used. These strategies are most often used separately in metabolomics studies, with each of them having their own limitations. We opted for a synergistic approach as a mean to unravel the most relevant metabolomics signature. We identified a set of nine metabolites, namely: nicotinamide, hypoxanthine, xanthine, and 1-methyl-6,7-dihydroxy-1,2,3,4-tetrahydroisoquinoline with decreased concentrations and N-acetyl-L-Leucine, arginine, RAC-glycerol 1-myristate, 1-oleoyl-RAC-glycerol, cystathionine with increased concentrations in POAG; the modification of nicotinamide, N-acetyl-L-Leucine, and arginine concentrations being the most discriminant. Our findings open up therapeutic perspectives for the diagnosis and treatment of POAG.

## 1. Introduction

Glaucoma is an age related disease characterized by the progressive loss of retinal ganglion cells, which are the neurons that transduce the visual information from the retina to the brain. It is the leading cause of irreversible blindness worldwide, with an incidence increasing due to population aging [1,2]. It is the World Health Organization’s priority in eye disease, known for the difficulties in providing an early diagnosis, its chronic evolution, and its large person-to-person difference in drug efficacy [3]. Glaucoma remains a poorly understood disease, with a range of therapeutic options targeting the main risk factor, the increased intra-ocular pressure, while there is currently no treatment to protect the retinal ganglion cells. 

Metabolomics is a powerful approach that enables the identification of metabolic signatures of pathological conditions, thus offering means for unraveling biomarkers and drug discovery. Metabolomics is the most recent field of the “omics” technologies that differs from other omics by taking into account the highly dynamic status of the biochemical phenotype of each individual, including genetics, environment, way of life, age, and sex factors [4], and thus better reflecting human phenotype, exposome, and clinical conditions than any other omics [5]. 

In this respect, we hypothesized that glaucoma will benefit from metabolomics investigations since only two studies performed on plasma from primary open-angle glaucoma (POAG) individuals were reported. The first one compared the plasma metabolome of POAG individuals (*n* = 72) with that of controls (*n* = 72), using a non-targeted metabolomics approach based on high resolution mass spectrometry. It revealed 41 discriminant metabolites including palmitoyl-carnitine, sphingolipids, terpenes, hydroxy-ergocalciferol, and other vitamin D-related molecules, thus indicating alterations of fatty acid, steroid, and sphingolipid metabolisms [6]. The second one, performed by our group, compared the metabolomics profiles of plasma from POAG individuals (*n* = 36) with age- and sex-matched controls with cataract (*n* = 27) using a targeted metabolomics approach. This study revealed 18 discriminant metabolites disclosing mitochondrial dysfunction with energetic substrates accumulation (hexoses and short- and medium-chain acyl-carnitines), senescence (increased phosphatidylcholines and decreased spermine, spermidine, and long-chain acyl-carnitines), and deficiency of polyamines (spermine and spermidine) known to be protective of retinal ganglion cells [7].

Herein, we report a new signature of POAG plasma using a non-targeted liquid chromatography coupled to a high resolution mass spectrometry (LC-HRMS) workflow, associated to a data mining strategy, which has recently proved its efficacy in the discovery of clinical biomarkers [8,9].

## 2. Results

The isotope labeled standards incorporated to the samples and quality controls (QCs) during sample preparation, served as quality control procedure. Isotope labeled standards variation in the QC and study samples were checked along the sequence to evaluate the retention time stability, the consistency of signal intensities, the mass accuracy within the sequence, and eventual drifts in instrumental sensitivity. These observations served as grounds for the acceptance of the analytical run.

Metabolomics profiling of the study cohort, i.e, 34 POAG patients and 30 age- and sex-matched controls, yielded 142 and 109 features with a coefficient of variation (CV) below 30% in positive and negative ionization modes, respectively. After manual curation, removal of isomers, and keeping only features detected in all samples, 95 metabolites in positive and 65 in negative modes were kept, thus leading to a final data matrix with 160 metabolites for statistical analyses. The PCA (principal component analysis) scatter plot showed clustering of pooled QC (quality control) samples as shown in Supplemental Figure S1, thus validating the sequence acquisition; no strong outlier was detected according to Hotelling’s T2 and DModX plots and no specific gender cluster was observed by PCA. 

Univariate analysis highlighted 28 metabolites with FDR (false discovery rate) below 5% (Table 1), among which six exhibiting a FC (fold change) greater than 1.5, i.e., nicotinamide, N-acetyl-L-leucine, hypoxanthine, cystathionine, 1-oleoyl-rac-glycerol, and 1-methyl-6,7-dihydroxy-1,2,3,4-tetrahydroisoquinoline. 

**Figure 1:**
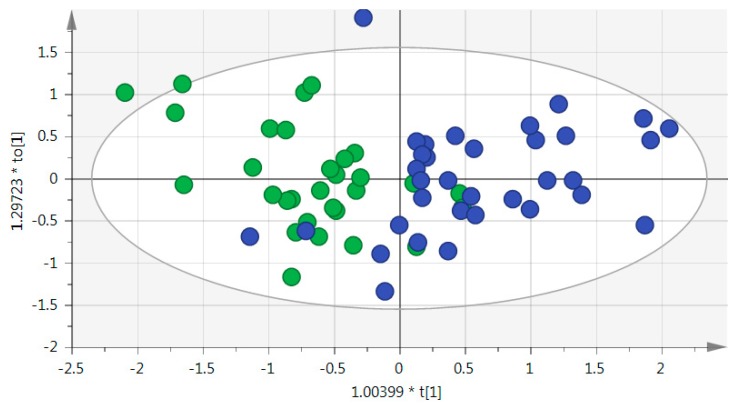
Score plot for the orthogonal partial least squares-discriminant analysis (OPLS-DA) model based on the 8 most significant metabolites from the S-plot. Control samples are represented by green circles; POAG (primary open-angle glaucoma) plasma is represented by blue circles. Goodness of fit (R2) Y (cum) = 0.5, goodness of prediction (Q2) (cum) = 0.4; coefficient of variation (CV) Anova *p*-value = 1.8042 × 10^−5^; Intercepts from the permutation test permR2 = 0.0355, permQ2 = −0.101.

The multivariate OPLS-DA (Orthogonal Partial Least Squares-Discriminant Analysis) model and S-plot (Supplemental Figures S2 and S3) revealed eight metabolites with variable importance in the projection (VIP) values greater than 1.3, which significantly contributed to class separation between glaucoma and control individuals (Figure 1, Table 1, Supplemental Table S1). This panel of metabolites included nicotinamide, N-acetyl-L-leucine, hypoxanthine, 1-methyl-6,7-dihydroxy-1,2,3,4-tetrahydroisoquinoline, 1-oleoyl-rac-glycerol, arginine, xanthine, and rac-glycerol 1-myristate, the latter three belonging to the metabolite panel highlighted by univariate analysis, but with a fold change of 1.3, hence, lower than 1.5. 

**Figure 2:**
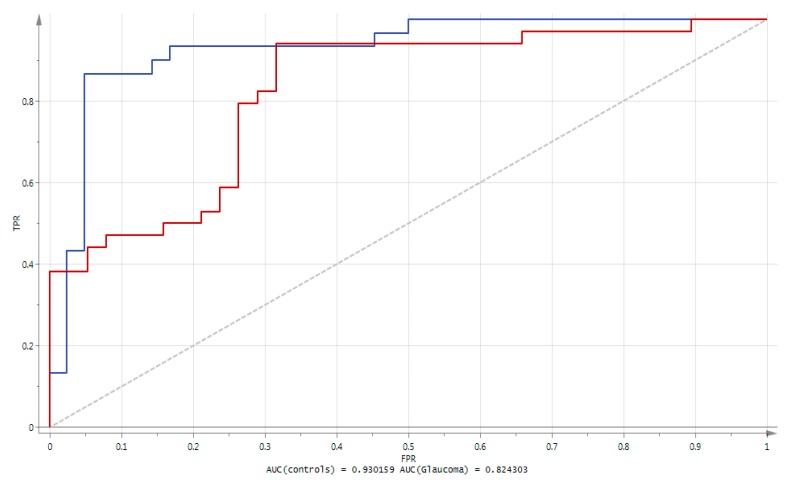
Results of the Receiver Operating Characteristic (ROC) curve analysis using the 8 metabolites derived from the multivariate OPLS-DA model. The blue line represents the Area Under the ROC Curve (AUC) obtained for controls, whilst the red line represents the AUC obtained for glaucoma patients. The ROC plots represent the sensitivity (i.e., True Positive Rate TPR) versus 1—the specificity (i.e., False Positive Rate FPR).

The prediction accuracy of the ROC (receiver operating characteristic) was 93.01% for controls and 82.43% for glaucoma individuals, using this panel of eight metabolites (Figure 2). 

This illustrates the performance of the metabolites of interest for correctly predicting non-affected and affected glaucoma individuals.

**Figure 3:**
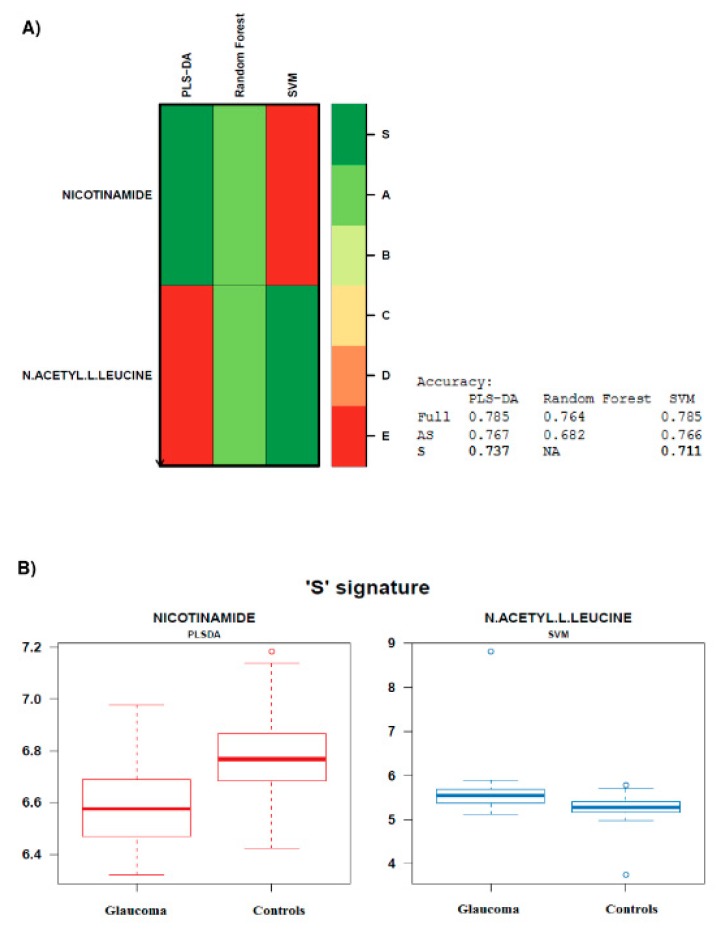
Biosigner signature. (**A**) The algorithm assessed the relevance of the 160 metabolites identified for the prediction performances of Partial Least Squares-Discriminant Analysis (PLS-DA), Random Forest (RF), and Support Vector Machines (SVM) classifier models and subsequently identified 2 robust ‘S tier’ features, i.e., nicotinamide and N-acetyl L-leucine. The accuracies of the PLS-DA and SVM models on the final S signature were respectively 73.7% and 71.1% for nicotinamide and N-acetyl L-leucine. (**B**) The boxplots depict the metabolite levels in glaucoma and control groups. Error bars represent ± s.e.m and the solid bars within the boxplots represent the median level of metabolite (log transformed peak area) for each group.

The restricted Biosigner algorithm suggested two ‘S’ tier metabolites, i.e., nicotinamide and N-acetyl-L-Leucine (Figure 3) from PLS-DA and Support Vector Machines (SVM) analysis, respectively. The accuracy of the model for the final S signature was superior to 70% for both metabolites. These two metabolites were also found to be significant by univariate and OPLS-DA analyses.

**Figure 4:**
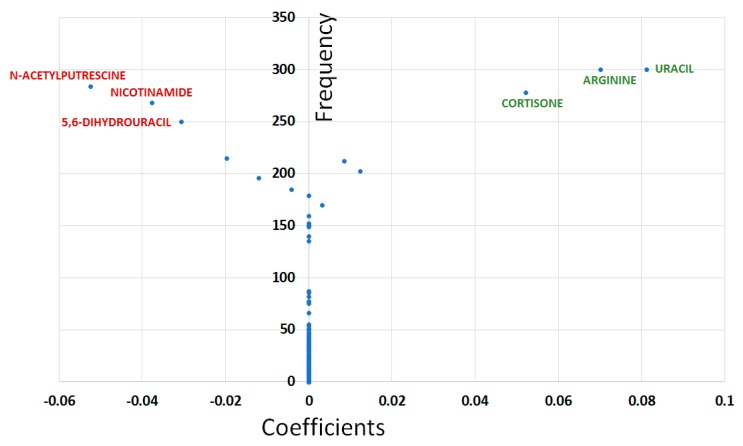
Least Absolute Shrinkage and Selection Operator (LASSO) Y‒plot displaying coefficients (x‒axis) and the number of models in which each variable appears (frequency, y‒axis) in the 303 models with highest predictive capabilities in the test set. Features from the first group show the highest absolute coefficient values. Positive and negative coefficients indicate relative higher and lower values for the corresponding feature in the glaucoma plasma compared to controls, and have been highlighted using green and red scriptures, respectively.

From the 1000 models generated by the Least Absolute Shrinkage and Selection Operator (LASSO) methodology on the training sets, 750 (75%) had an area under the ROC curve (AUC) ≥ 0.8 when applied to the test sets; the median and mean values for AUC being 0.86 and 0.84, respectively (Supplemental Figure S4). Three hundred and three (30.3%) models were used in variable selection as they exhibited very good predictive performances on the test set (AUC ≥ 0.9). Plotting the frequency of each feature in the predictive model enabled the identification of three groups of variables: I—features selected in at least 250 models (six variables: uracil, arginine, N-acetyl-putrescine, nicotinamide, cortisone, and 5,6-dihydrouracil); II—features selected in less than 250 but in more than 100 models (13 variables), and III—features selected in less than 100 out of the 303 best predictive models (141 variables) (Supplemental Figure S5). The Y-plot shows that variables from the first group had the highest absolute coefficients, whilst variables from the third group had, as expected null, median coefficients (Figure 4). 

Among the group I variables identified following LASSO, uracil, N-acetyl-putrescine, and cortisone have a VIP value less than 1.3, whilst 5,6-dihydrouracil has a non-significant *q*-value (*q* = 0.0986). Accordingly, we kept only nicotinamide and arginine as the main features for the LASSO model (Table 1).

**Figure 5:**
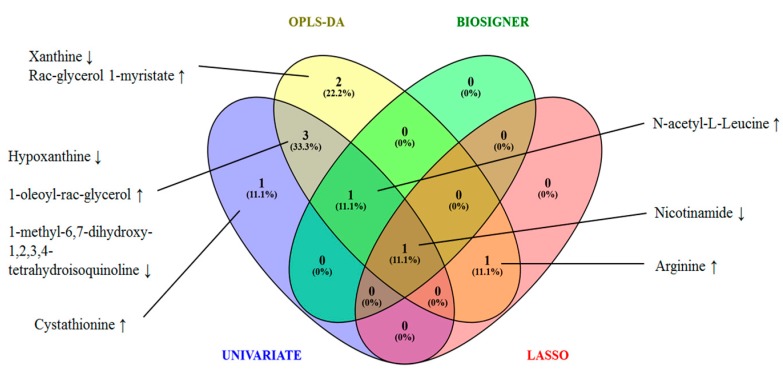
Venn diagram illustrating the global POAG signature.

To summarize the results obtained from the statistical analyses, we constructed a Venn diagram providing an overview of the global POAG signature (Figure 5). We used strict selection criteria, which provide a good compromise between prediction accuracy and signature stability. In this respect, the POAG signature includes nine significant metabolites: nicotinamide, N-acetyl-L- leucine, arginine, hypoxanthine, xanthine, Rac-glycerol 1-myristate, 1-oleoyl-rac-glycerol, cystathionine, and 1-methyl-6,7-dihydroxy-1,2,3,4-tetrahydroisoquinoline. The biomarkers reported in this research were allocated to the identification level 1, according to the current metabolomics standards initiative (MSI) reporting standards [10]. 

Furthermore, in the context of biomarker discovery useful for clinical diagnosis, the selection of a restricted list of candidate markers is required before entering a subsequent qualification and verification phase [11,12,13]. In this respect, as detailed elsewhere [14], we proceeded to a blind independent external validation of nicotinamide, the metabolite identified by all four statistical approaches. Nicotinamide dosage using an independent quantitative method was performed on plasma samples from both the initial cohorts including 64 individuals reported in the current investigation, and replicative cohorts of 35 individuals. Nicotinamide quantification confirmed the observation made from the non-targeted metabolomics analysis reported here.

## 3. Discussion

In the absence of an exhaustive method to explore the whole metabolome, combinations of different approaches are required to progress the deciphering the extensive blood metabolic signature of glaucoma. To complete the two studies previously published [6,7] that evidenced 41 and 18 discriminant metabolites, using non-targeted and targeted metabolomics, respectively, we here carried out a non-targeted method, allowing the precise identification of 500 metabolites [8]. Among the 160 metabolites accurately detected using this approach, univariate analysis highlighted 28 discriminant metabolites, with FDR below 5%, whereas the combination of multivariate analysis, LASSO tests and machine learning approaches restricted this list to the nine most discriminating metabolites. Importantly, 25/28 (univariate) and 8/9 (multivariate) discriminant metabolites reported here are novel metabolites related to POAG signature. 

Nicotinamide deficiency was the most important feature, supported by its identification by the four data analyses performed in this study (Table 1). This feature represents a special interest for glaucoma treatment. Indeed, a study of the mouse DBA/2J model with high intra-ocular pressure has recently established the proof of concept of vitamin B3 therapeutic interest [15] to prevent glaucoma. Our current investigations in humans [7] together with the recent report in mice, argues in favor of a mitochondrial-related nicotinamide deficiency, contributing to the age-related vulnerability of the optic nerve in glaucoma pathogenesis. This statement is further reinforced by the fact that mitochondrial complex I deficiency was also reported in lymphoblasts from glaucoma patients [16], a fact that could be related to the decrease level of nicotinamide adenine dinucleotide (reduced form) (NADH) in these cells [17]. 

The increased concentration of N-acetyl-L-Leucine is the second most important discriminating feature, evidenced by univariate analysis, OPLS-DA analysis, and the Biosigner algorithm. This molecule is widely prescribed as an anti-vertigo drug (Tanganil^®^) that facilitates the repolarization of vestibular neurons [18] and is known for its endogenous neuroprotective activity in mammals [19]. Only one patient in our glaucoma cohort used Tanganil and none in the control cohort, thus excluding an effect related to a systemic medication with N-acetyl-L-Leucine. Understanding its higher concentration in glaucoma remains to be addressed. 

The increased arginine concentration in POAG plasma was evidenced by three of the four data methods and was already reported in the targeted metabolomics study [7], together with that of methionine and tyrosine, which were also identified here by the univariate analysis, thus attesting the reproducibility of the results obtained with two different mass spectrometry approaches. Modification of arginine concentration may be related to the altered arginine/nitric oxide regulatory pathway that characterize glaucoma [20]. The 1-methyl-6,7-dihydroxy-1,2,3,4-tetrahydroisoquinoline, also known as salsolinol, a endogenous derivate of dopamine, was decreased in POAG patients. Its activity inhibits tyrosine hydroxylase, which would explain the increased concentration of tyrosine in the glaucoma signature. 

Hypoxanthine and xanthine concentrations are also reduced in POAG signature. Interestingly, we found similar decreased concentrations of hypoxanthine and xanthine in the plasma of patients with Dominant Optic Atrophy related to *OPA1* mutations [9]. These findings argue in favor of a mitochondrial impairment that contribute to glaucoma pathogenesis, taking into account the role played by mitochondria in nucleotide metabolism and the similitude with primary mitochondrial diseases leading to optic neuropathy. 

Further, two monoacylglycerides, i.e., RAC-glycerol-1-myristate, also known as monomyristin, and 1-oleoyl-RAC-glycerol, also known as monolein, were significantly increased here, but without clear rationale yet to be established. Cystathionine, also modified in the POAG signature, is metabolically tightly connected to methionine and cysteine through sulfur, thiols, and folate metabolisms, which were already incriminated in glaucoma [21,22].

The POAG signature also disclosed alterations of the monosaccharides’ metabolism, with galactose and arabinose displaying increased concentrations in patients vs. controls. These results support the findings from targeted metabolomics investigations in which the sum of hexoses was significantly increased in POAG plasma [7]. The increased concentration of glyceraldehyde, an intermediate of glycolysis also argues in favor of carbohydrate metabolism perturbations, paralleling previous results reporting alterations of galactose, fructose, and mannose metabolisms in the plasma of POAG patients [6]. 

One limitation of our study is that it was only carried out on a single cohort of patients and controls. Other studies of this type will be necessary to define whether the metabolomic characteristics presented here can be generalized to all patients with glaucoma. However, in order to validate the nicotinamide deficiency, we previously measured the level of this vitamin using a targeted LC-MS/MS method specifically designed for the quantification of nicotinamide. The cohort of the current non-targeted study (34 POAG patients and 30 controls), as well as a replication cohort comprising 20 POAG patients and 15 controls were both analyzed [14]. Nicotinamide deficiency observed during the present non-targeted LC-HRMS metabolomics investigation was confirmed by targeted LC-MS/MS in both the initial cohort and the replication cohort, thus strengthening the results obtained in the present study. A second limitation of our study lies in the difficulty of stratifying the glaucoma phenotype in relation to other potential comorbidities in this population of elderly patients. Although no comorbidity other than glaucoma significantly distinguishes our POAG population from that of controls, we cannot completely exclude the influence of other pathologies on the metabolomics signature we report here.

## 4. Materials and Methods 

### 4.1. Chemicals and Reagents 

Solvents and reagents used in sample preparation and mobile phase were of Optima LC-MS-grade. Methanol (MeOH), water (H_2_O), and formic acid were purchased from Fisher Scientific (Illkirch, France). Isotope labeled endogenous metabolite standards (at a minimal purity of 98%), namely 17α-Hydroxyprogesterone-d8 (2,2, 4,6,6,21,21,21-d8), L-Thyroxine-13C6, Succinic acid-2,2,3,3-d4, Pyruvic acid-1-13C, and DL-Alanine-15N were purchased from Sigma Aldrich (St. Quentin Fallavier, France). A working solution at a concentration of 10 μg/mL of each isotope labeled standards was prepared in MeOH.

### 4.2. Ethics Statement

Participants were included in the study after giving their informed written consent for the research, which was conducted according to the ethical standards of the Declaration of Helsinki (1983), and with the approval of the local Ethical Committee (Comité de Protection des Personnes (CPP) OUEST 2), agreement number: CB 2013-04. 

### 4.3. Study Cohorts

The study cohorts were composed of 34 POAG and 30 age- and sex-matched control individuals whose recruitment criteria and clinical features were recently described in a study focused on a quantitative dosage of nicotinamide [14]. Briefly, the individuals were recruited from the Department of Ophthalmology of Angers University Hospital, France. The diagnosis of POAG was based on consensual criteria, i.e., intraocular pressure >21 mmHg, and glaucomatous optic nerve damage with progressive optic disc cupping [23]. Individuals with isolated ocular hypertension, normal tension glaucoma, or any secondary form of glaucoma, were excluded from the study. 

Control subjects were selected among healthy individuals undergoing cataract surgery at the same Department of Ophthalmology. Their inclusion criteria were visual acuity ≥20/50 and the absence of any other associated ocular condition, excepting cataract. Exclusion criteria were a family history of glaucoma, ocular hypertension, or any other intraocular pathology, including retinal disorders. 

As previously detailed, there was no significant difference between the two groups in terms of mean age, sex ratio, body mass index, diabetes, hypertension, hyperlipidemia, thyroid disease, systemic medications, and mean intraocular pressure (IOP) [14]. POAG patients had an elevated IOP at the time of initial diagnosis. However, at the time of inclusion, these patients had been treated, which explains there was no difference in IOP compared to controls.

### 4.4. Plasma Sample Collection and Metabolites Extraction

Blood samples from each participant were collected in heparin tubes at least three hours after the last meal. The transfer of the blood tubes was carried out according to a strict protocol, securing the fastest possible storage at −80 °C. Thus, after blood sampling, the tubes were immediately transported on ice to the certified Biological Resource Center (Hospital of Angers), where they were immediately processed for centrifugation (10 min at 2500 × *g* at +4 °C) to recover the supernatant (plasma), which was aliquoted in 500 microliter aliquots, and immediately stored at −80 °C until analysis. The delay between sampling and storage was less than one hour for every included subject. Metabolites extraction was performed following the procedure previously reported [8]. Briefly, plasma samples were allowed to thaw on ice and sample deproteinization was achieved using ice-cold methanol (−20 °C). Two hundred and sixty microliter of cold MeOH was added to 30 μL plasma and fortified with 10 μL of isotope labeled endogenous metabolite solution (10 μg/mL of each). The mixture was vortexed for 30 s and centrifuged (8000 × *g*, 10 min, 4 °C). The supernatant was then collected and evaporated in a miVac duo concentrator (Genevac Ltd., Ipswich, United Kingdom). The dry extract was reconstituted with 200 μL of a solution of 2% aqueous MeOH (initial conditions of the chromatographic elution gradient). The mixture was subjected to a second centrifugation (8000 × *g*, 10 min, 4 °C) to remove fine particles prior to reverse phase (RP) liquid chromatography (LC)-high resolution mass spectrometry (HRMS) analysis. A pooled quality control (QC) sample deriving from the study population was prepared. QC samples were extracted along with the study cohort and analyzed throughout the analytical run. QC dilution series (1, 1/2, 1/4) were also carried out to provide robust quality assurance for each metabolic feature detected.

### 4.5. Metabolomics Profiling

Metabolic profiling benefited from the separation and sensitive detection provided by the Ultra High Performance Liquid Chromatography (UHPLC, Dionex™ UltiMate 3000, ThermoFischer Scientific, Waltham, MA, USA), coupled to a High Resolution Mass Spectrometer (HRMS) Thermo Scientific™ Q Exactive™ platform. Acquisitions were performed in positive (HESI+) and negative (HESI–) ionization modes as previously reported [8]. 

Accurate *m/z* values, retention times, isotopic patterns, full MS and MS/MS fragmentation spectra were acquired for each metabolite, to facilitate identification. The TraceFinder 4.1 software was used for metabolites identification and peak integration using the in-house library. The data matrix containing identified features was further filtered out based on the following criteria: the coefficient of variation (CV) below 30%, accurate *m/z* measurement with delta ppm < 5, isotopic pattern (masses and abundances of the isotopes matching the chemical formula, pass), expected retention time (RT, pass), linearity for dilutions 1, 1/2, and 1/4 (r^2^ ≥ 0.8), library search (pass), and MS/MS fragments (Top5 ddMS2 experiments) database matching. Estimation of RT drift was made from RT of the metabolite in the internal library vs. experimental RT of metabolite during the sequence; ΔRT ± 10 s was used. The data matrix was manually checked, metabolites exhibiting no missing values for the entire patient cohort were kept, and redundancies (isomers) were removed.

The recommendations of the metabolomics standards initiative (MSI) [10] were considered during this research.

### 4.6. Statistical Analyses and Features Selection

In order to identify key metabolites responsible for the differential status of the study cohorts, a combination of univariate and multivariate statistical methods were used. Before univariate and multivariate statistics, the data matrix generated from TraceFinder preprocessing was normalized to take into account potential instrumental drift which may occur when processing a large number of samples; the LOESS (Locally weighted Scatterplot Smoother) regression function available on Galaxy Workflow4Metabolomics [24] was used to this end (galaxy.workflow4metabolomics.org (in the public domain)). This process makes samples comparable. Indeed, the normalization provided correction of analytical effects on metabolite intensities within the batch, using pooled quality control (QC) samples that ran throughout the analytical sequence.

The resulting data matrix was subjected to log transformation and Pareto scaling. Log-transformation improved variance homogeneity, while Pareto-scaling, a useful compromise between unit variance-scaling and no scaling, enhances the contribution from medium sized features without increasing the chemical noise. 

A data mining strategy was further performed to unveil the most relevant glaucoma biomarkers. The strategy combined the filtering method with threshold criterion, the wrapper method with iterative selection, and the embedded method with penalization constraint, as detailed below. These strategies are most often used separately in metabolomics studies for features selection. Each approach having its own limitation, we opted for a synergistic approach with the rationale to extract a molecular signature demonstrating the best prediction performance (sensitivity, selectivity), stability (reproducibility), and relevance. 

The data matrix was subsequently subjected to the following statistical analyses:

(1) Principal component analysis (PCA) was employed to provide an overview of the population structure and to ensure clustering of the pooled quality controls (QCs). Hotelling’s T2 and DModX plots were visually inspected for detecting outliers. The analysis was performed using SIMCA-P v.14.0 (Umetrics, Umea, Sweden);
(2) Univariate analysis was performed using the non-parametric Wilcoxon rank sum test with Benjamini–Hochberg correction and keeping the False Discovery Rate (FDR) below 5%. Features were further filtered according to their fold change (FC) and only metabolites with a FC greater than 1.5 were further considered. These analyses were conducted using Metaboanalyst v4.0 [25];
(3) Orthogonal Projection of Latent Structures-Discriminant Analysis (OPLS-DA) was subsequently performed. The method offers a convenient way of explicitly taking into account the class membership of observations, and to visualize metabolites responsible of the class separation (in this case glaucoma vs. controls). This was followed by an S-plot which provided the visualization of variables’ influence in the OPLS-DA model. Selection of putative biomarkers from the S-plot was combined to jack-knife confidence intervals from a loading column plot; features were further filtered according to their variable importance in the projection (VIP) and only metabolites with VIP values greater than 1.3 were further considered. Consequently, only metabolites with strong model contribution and highly statistical reliability were selected as discriminated metabolites (putative biomarkers). The quality of the finally obtained OPLS-DA model was evaluated by R2 (goodness of fit, i.e., how well the model fits the data), Q2 (goodness of prediction, i.e., how well the model predicts new data) parameters, cross validation-analysis of variance CV-ANOVA and a permutation test. The performance of the identified metabolites was assessed using area under the ROC curve (AUC). The ROC (receiver operating characteristic) can be understood as a plot of the probability of correctly classifying the positive samples against the rate of incorrectly classifying true negative samples. The AUC measure of a ROC plot is a measure of predictive accuracy. A classifier with a perfect discrimination has a ROC curve that passes through the upper left corner (100% sensitivity, 100% specificity); conversely, a ROC curve close to the 1:1 diagonal represents a very poor classifier. These analyses were performed using SIMCA-P v.14.0 (Umetrics, Umea, Sweden);
(4) Biosigner signature was obtained using the new wrapper algorithm ‘Biosigner’ [26]. The algorithm is wrapped around three machine learning approaches ran in parallel, i.e., PLS DA, Random Forest (RF), and Support Vector Machines (SVM). It is an iterative algorithm aiming at the selection of the most promising candidates, i.e., providing a signature of restricted size, of high stability, and high prediction accuracies. The wrapper algorithm is based on random permutation of feature intensities in test subsets obtained by resampling, to assess the significance of the features on the model performance by dichotomy. The S tier corresponds to the final signature, i.e., significant metabolites, which passed all the selection iterations. Biosigner module implemented in Galaxy Workflow4Metabolomics [24] was used, with the aim of finding the smallest feature subset which significantly contributes to the model performance, and optimally discriminates between glaucoma and control individuals. The advanced computational parameters were used, with the following settings: number of bootstraps: 50, selection tier: S, *p*-value threshold: 0.05, and seed: 1;
(5) The Least Absolute Shrinkage and Selection Operator (LASSO) method was performed using the R package glmnet. LASSO combines feature selection and model construction in a single step, by including a penalization constraint within the algorithm; the embedded approaches limit the number of features with non-zero coefficients in the final model [27]. LASSO analysis reveals features that assisted in unambiguous class separation. Before performing LASSO, all data were divided into training (~2/3) and test sets (~1/3). One thousand different training and test sets were created by randomly allocating each patient and control to either the training or the test set. The LASSO method was performed on each training set and the model obtained was then applied to the corresponding test set to evaluate the predictive capabilities of the model. The AUC was used to evaluate the predictive performance of the LASSO model on the test set. Models yielding AUC ≥ 0.8 on the test sets were considered as good models whilst the predictive capabilities of those having AUC ≥ 0.9 were considered as excellent. When the LASSO method displayed good general predictive capabilities (median AUC ≥ 0.8), variable selection was performed by measuring the frequency (F) at which each feature was selected (i.e., non-null coefficient) in models having excellent predictive capabilities when applied to the test sets (i.e., AUC ≥ 0.9). Models with AUC ≥ 0.9 in the test sets were used to estimate metabolite coefficients (C) calculated as the median values of coefficients obtained in each of these models with excellent predictive capabilities. Plotting F vs. C yields a Y-shape graph (called “Y-plot” here) with the vertical branch of the Y accounting for the variables having null median coefficients and left and right upper branches reflecting most likely important variables with decreased (respectively increased) metabolite concentrations in the glaucomatous condition compared to controls.

A Venn diagram highlighting common and distinct metabolites between the four statistical strategies (univariate analysis with FDR correction and FC greater than 1.5, OPLS-DA with VIP values greater than 1.3, Biosigner and LASSO methods) was built. The diagram was drawn using Venny 2.1 

## 5. Conclusions

In conclusion, the data presented here raised special interest in a new set of metabolites that partially confirm previous non-targeted and targeted metabolomics studies, and enrich the list of metabolites that are affected in POAG. Taken together, these three studies converge towards several metabolic pathways strongly implicated in glaucoma, such as disruption of amino acids metabolism, principally arginine, tyrosine, and methionine; disruption of energetic supply (fatty acids and monosaccharides), age-related mitochondrial defect (nicotinamide), impairment of nucleotide metabolism (xanthine and hypoxanthine), and the involvement of the endogenous neuroprotective N-acetyl-L-Leucine. Further studies will be necessary to unravel the role of these metabolic pathways in the different forms and severity of glaucoma. Lastly, the detailed data analysis workflow, combining several statistical and machine learning methods introduced here, is valuable. It facilitates data filtering and further leads to the most relevant metabolomics signature.

## Figures and Tables

**Table 1:** Metabolites selection from the 4 statistical analyses.

Metabolites	Univariate Analysis			OPLS-DA	BIOSIGNER	LASSO
	^a^ Fold Change (POAG/Control)	^b^ FDR *q*-Value	VIP Values			
Nicotinamide *	0.643 ↓	0.00269	2.06794	*√*	*√*	*√*
Arginine	1.312 ↑	0.00375	1.36764	*√*		*√*
Glyceraldehyde	1.106 ↑	0.00375	0.94874			
N-acetyl-L-leucine *	1.846 ↑	0.00375	2.02646	*√*	*√*	
Galactose	1.103 ↑	0.00444	0.93104			
Hypoxanthine *	0.558 ↓	0.00444	3.13134	*√*		
1-methyl-6,7-dihydroxy-1,2,3,4-tetrahydroisoquinoline *	0.469 ↓	0.00444	2.61827	*√*		
Glyoxylic acid	1.097 ↑	0.00523	0.90817			
Arabinose	1.121 ↑	0.00523	0.98914			
Xanthine	0.727 ↓	0.00629	1.95504	*√*		
3-hydroxybenzaldehyde	1.325 ↑	0.00938	1.12595			
Tyrosine	1.213 ↑	0.01127	1.30588			
Indole-3-acetate	1.449 ↑	0.01791	1.36766			
Urocanate	0.733 ↓	0.01791	1.58454			
Uracil	1.048 ↑	0.02246	0.42581			
N-acetylputrescine	0.812 ↓	0.02285	1.24824			
3-hydroxyphenylacetate	1.398 ↑	0.02285	1.37148			
Glycolate	1.13 ↑	0.02285	0.84268			
Rac-glycerol 1-myristate	1.316 ↑	0.03136	1.63356	*√*		
Methionine	1.163 ↑	0.03268	1.27457			
Alpha-aminoadipate	1.306 ↑	0.03409	1.59113			
Cystathionine *	1.656 ↑	0.03886	2.46291			
Cortisone	1.35 ↑	0.04246	0.88469			
Uridine	0.811 ↓	0.04246	1.41792			
cis-4-hydroxy-d-proline	1.443 ↑	0.04444	1.32333			
4-hydroxy-l-proline	1.432 ↑	0.04463	1.32638			
Cystine	0.841 ↓	0.04487	1.0005			
1-oleoyl-rac-glycerol *	1.608 ↑	0.04906	1.53561	*√*		

Univariate analysis highlighting the 28 significant metabolites after correction of the False Discovery Rate (FDR) threshold, sorted by decreased *q*-values. * Metabolites in italic display a fold change (FC) greater than 1.5. ^a^ FC were calculated using median values, as the ratio of primary open-angle glaucoma (POAG) group to control group. ^b^ *q*-values were calculated from a non-parametric Wilcoxon rank sum test with Benjamini–Hochberg correction and keeping the FDR below 5%. √ Metabolite found discriminant using the test. OPLS-DA: Orthogonal Partial Least Squares-Discriminant Analysis; LASSO: Least Absolute Shrinkage and Selection Operator; ↑: increased concentration in POAG compared to controls; ↓: decreased concentration in POAG compared to controls.

## Supplementary Materials

The following are available online at https://www.mdpi.com/2218-1989/10/2/49/s1, Figure S1: PCA applied to the 160 identified features in the initial cohort, Figure S2: Score plot for Orthogonal Projection of Latent Structures Discriminant Analysis (OPLS-DA) model based on the entire dataset, Figure S3: S-plot for biomarkers selection, Figure S4: Histogram of the area under the ROC curve (AUC) versus the number of LASSO models, Figure S5: LASSO model, Table S1: Data matrix after curation.
