# Supplementary material for: A Data Mining Metabolomics Exploration of Glaucoma
Source: Metabolites. 2020 Jan 28;10(2):49. doi: 10.3390/metabo10020049 (PMC7074047; doi:10.3390/metabo10020049)
Supplement: Supplementary file 1 [file metabolites-10-00049-s001.pdf]

A data mining metabolomics exploration of glaucoma

Judith Kouassi Nzoughet [1,2,*], Khadidja Guehlouz [3], Stéphanie Leruez [3], Philippe Gohier [3], Cinzia Bocca [1], Jeanne Muller [3], Odile Blanchet [4], Dominique Bonneau [1,5], Gilles Simard [5], Dan Milea [6], Vincent Procaccio [1,5], Guy Lenaers [1], Juan M. Chao de la Barca [1,5], Pascal Reynier [1,5]

[1] Faculté de santé, Institut MITOVASC, UMR CNRS 6015, INSERM U1083, Université d'Angers, Angers, France
[2] Faculté de Pharmacie de Paris, CiTCoM UMR 8038 CNRS, Université Paris Descartes, Paris, France
[3] Département d'Ophtalmologie, Centre Hospitalier Universitaire, Angers, France
[4] Centre de Ressources Biologiques, BB-0033-00038, Centre Hospitalier Universitaire, Angers, France
[5] Département de Biochimie et Génétique, Centre Hospitalier Universitaire, Angers, France
[6] Singapore Eye Research Institute, Singapore National Eye Centre, Duke-NUS, Singapore.

* Correspondence: judith.nzoughet-kouassi@parisdescartes.fr (J.K.N.); pareynier@chu-angers.fr (P.R.)

Supplemental Figures

Supplemental Figure S1……………………………………………………………Page S2

Supplemental Figure S2……………………………………………………………Page S3

Supplemental Figure S3……………………………………………………………Page S4

Supplemental Figure S4…………………………………………………………....Page S5

Supplemental Figure S5…………………………………………………………....Page S6

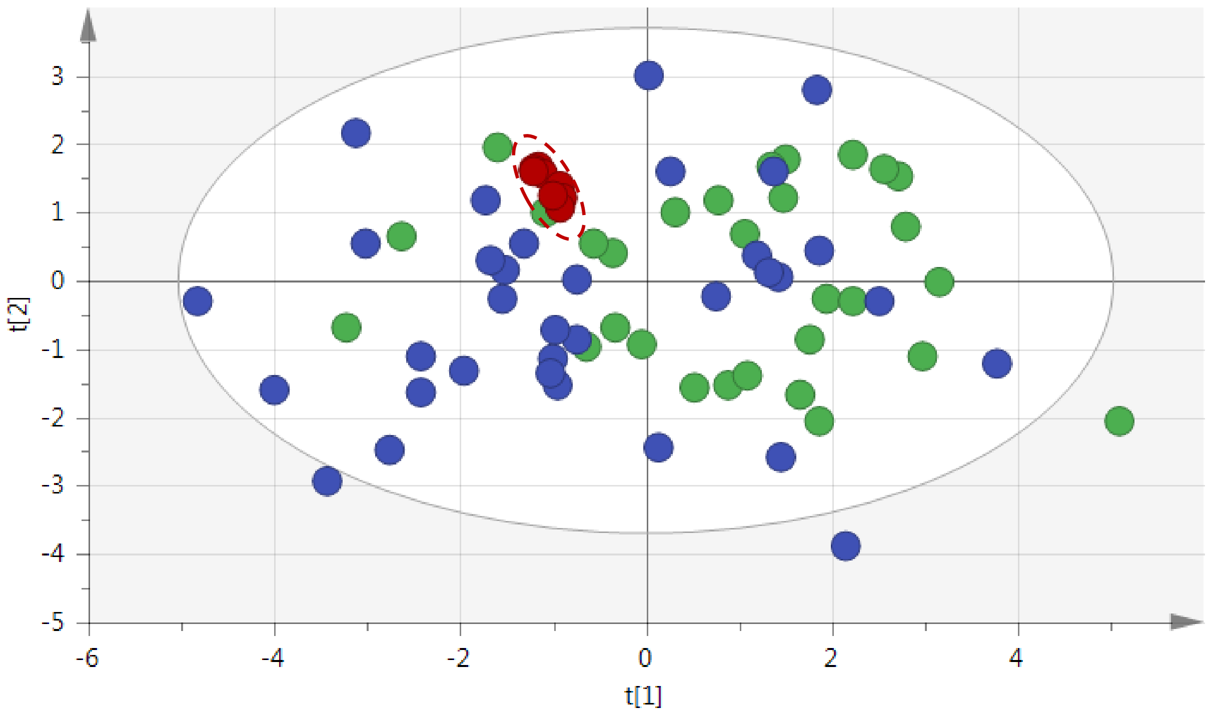

Supplemental Figure S1. PCA applied to the 160 identified features in the initial cohort, including 34 POAG patients (*blue circles*), 30 age- and sex-matched controls (*green circles*) and 8 QC samples (*red circles*). PCA scatter plot shows clustering of pooled QCs.

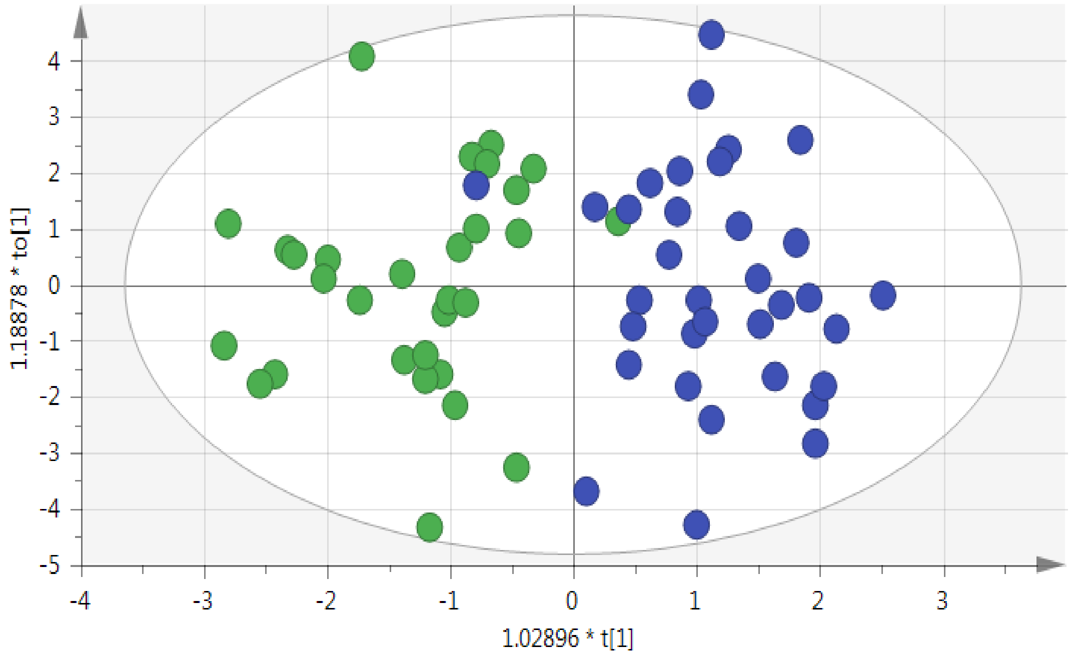

Supplemental Figure S2. Score plot for Orthogonal Projection of Latent Structures Discriminant Analysis (OPLS-DA) model based on the entire dataset, including 160 metabolites and 64 individuals. Control samples are represented by *green circles*; glaucoma patients are represented by *blue circles*. R2Y(cum) = 0.7, Q2 (cum) = 0.5; CV Anova p-value= 2.8e-007 ; Intercepts from the permutation test permR2= 0.053, permQ2= -0.226.

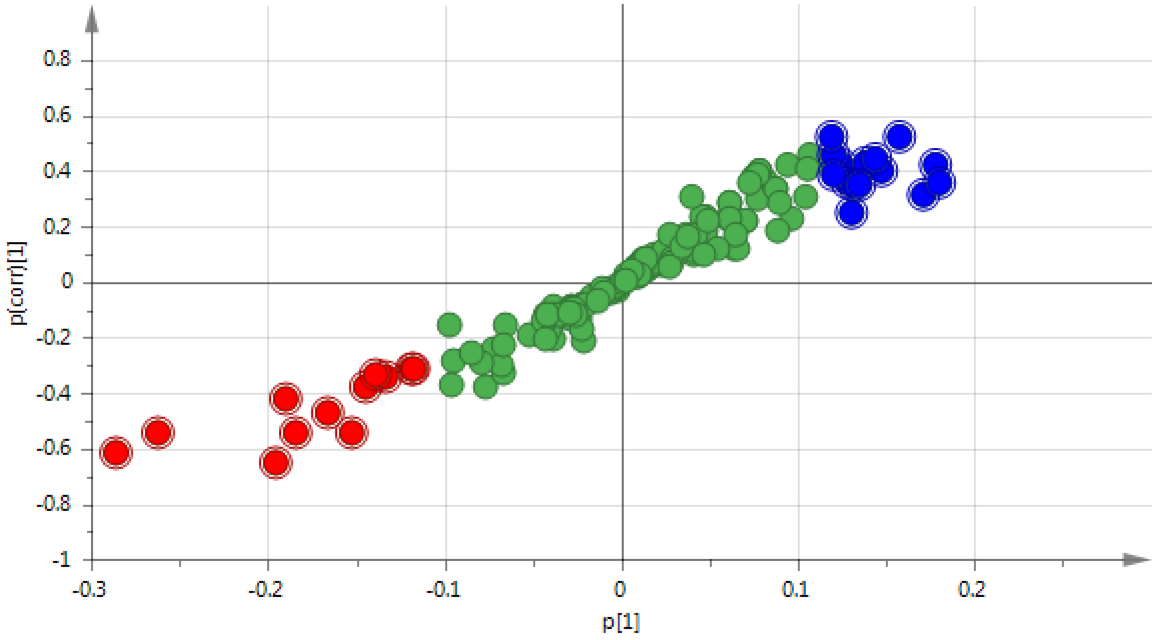

Supplemental Figure S3. S-plot for biomarkers selection; the representation combines the covariance (*p* representing the magnitude – modeled covariation) and the correlation (*pcorr* representing the reliability – modeled correlation) loading profiles. The S-plot derived from the OPLS-DA model based on the entire dataset. We focused on the blue-highlighted (up-regulated metabolites ↑) and red-highlighted (down-regulated metabolites ↓) features and considered those meeting the selection criteria. A panel of 8 metabolites was further selected.

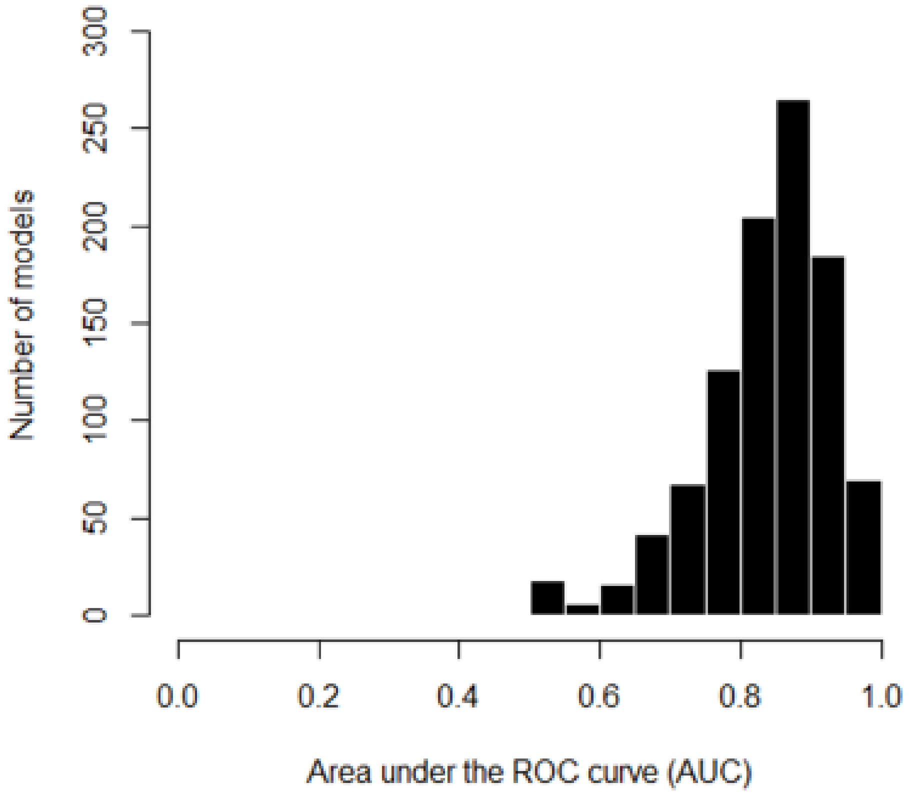

Supplemental Figure S4. Histogram of the area under the ROC curve (AUC) versus the number of LASSO models. The AUC of 750 models out of 1000 models (75%) is at least 80%. Median and mean AUC for the 1000 models was 0.86 and 0.84, respectively.

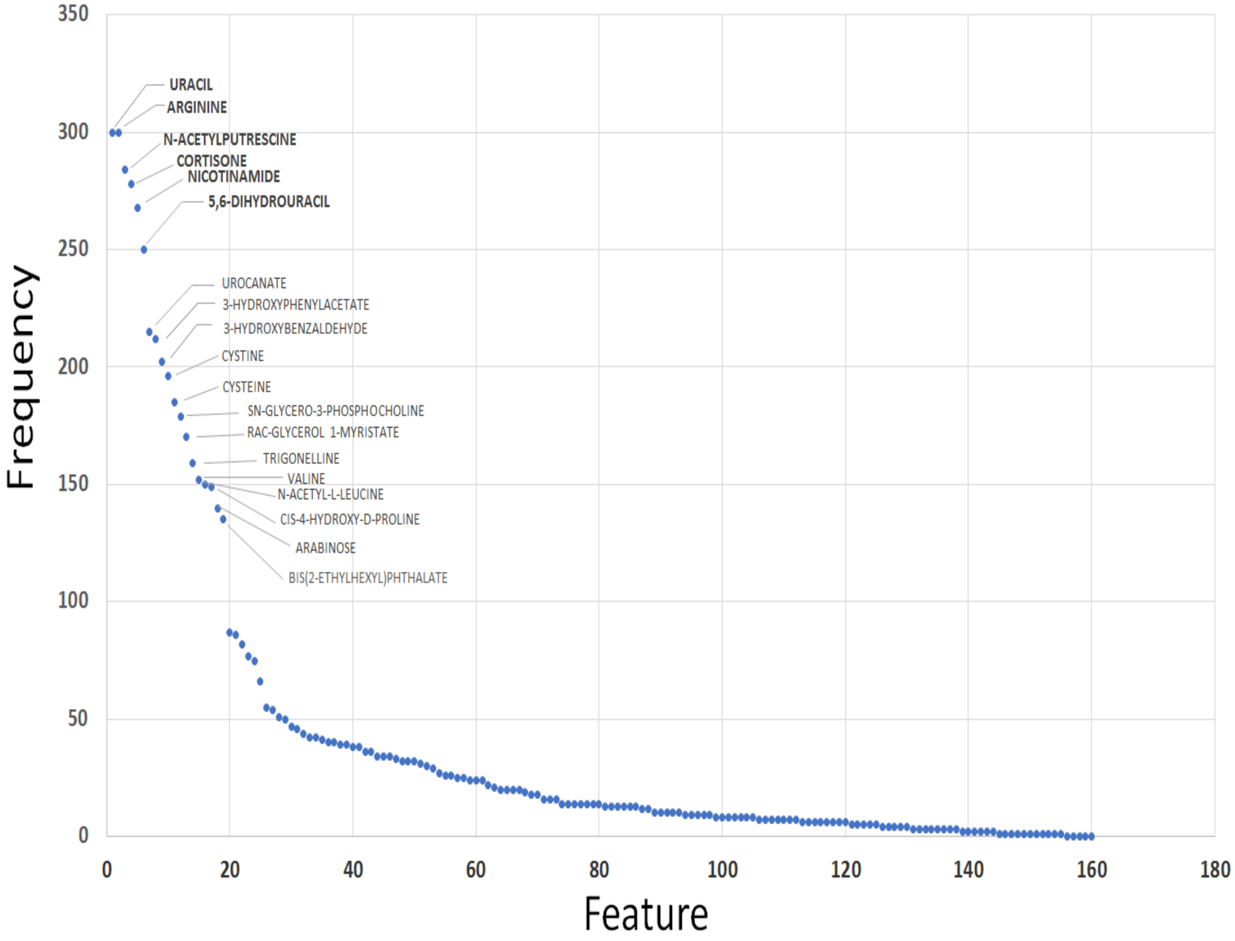

Supplemental Figure S5. LASSO model; three groups of variables are easily distinguishable according to the frequency they were kept in the 303 models with excellent predictive capabilities. The first group includes 6 features (uracil, arginine, N-acetyl-putrescine, cortisone, nicotinamide and 5,6-dihydrouracil) that were kept in at least 250 models (82.5%); the second group represents features selected in less than 250 but in more than 100 models (13 variables) and the third group contains features that were kept in less than 100 models (~ 33%). Variables in the first group have been labelled using bold font and variables from the third group have not been labelled.
